# Heterogeneity in coverage for measles and varicella vaccination in toddlers – analysis of factors influencing parental acceptance

**DOI:** 10.1186/s12889-017-4725-6

**Published:** 2017-09-19

**Authors:** Christine Hagemann, Andrea Streng, Alexander Kraemer, Johannes G. Liese

**Affiliations:** 1 Department of Pediatrics, University of Würzburg, Josef-Schneider-Str. 2, 97080 Würzburg, Germany; 2 Department of Public Health Medicine, School of Public Health, University of Bielefeld, Universitätsstr. 25, 33615 Bielefeld, Germany

**Keywords:** Varicella, Measles, Vaccination, Coverage, Surveillance, Pediatric

## Abstract

**Background:**

In 2004, routine varicella vaccination was introduced in Germany for children aged 11–14 months. Routine measles vaccination had already been introduced in 1973 for the same age group, but coverage is still too low (<95%) in some areas to eliminate measles. The present study assessed varicella and measles vaccination coverage and determinants of parental acceptance in two study regions, situated in Northern and Southern Bavaria (Germany).

**Methods:**

From 2009 to 2011, annual cross-sectional parent surveys were performed on random samples of 600 children aged 18–36 months in the Bavarian regions of both Munich and Würzburg. Logistic regression models were used to identify factors associated with varicella and measles vaccination.

**Results:**

In 2009, 2010 and 2011, vaccination coverage was lower in Munich than in Würzburg, for both varicella (Munich 53%, 67%, 69% vs. Würzburg 72%, 81%, 83%) and for measles (Munich 88%, 89%, 91% vs. Würzburg 92%, 93%, 95%). Recommendation by the physician was the main independent factor associated with varicella vaccination in both regions (adjusted odd ratios (OR) with 95% confidence interval (CI): Munich OR 19.7, CI 13.6–28.6; Würzburg OR 34.7, CI 22.6–53.2). Attendance at a childcare unit was positively associated with a higher acceptance of varicella vaccination in Munich (OR 1.5, CI 1.1–2.2). Regarding measles vaccination, attendance at a childcare unit was positively associated in both regions (Munich OR 2.0; CI 1.3–3.0; Würzburg OR 1.8; CI 1.1–3.1), and a higher level of parental school education was negatively associated in Würzburg (OR 0.5, CI 0.3–0.9).

**Conclusions:**

Vaccination rates differed between regions, with rates constantly higher in Würzburg. Within each region, vaccination rates were lower for varicella than for measles. Measles vaccination status was mainly dependent upon socio-demographic factors (attendance at a childcare unit, parental school education), whereas for the more recently introduced varicella vaccination recommendation by the physician had the strongest impact. Hence, different strategies are needed to further improve vaccination rates for both diseases.

## Background

Varicella (chickenpox) was one of the most widespread diseases in Germany during the pre-vaccination era, with an estimated incidence of 9.3 per 1000 inhabitants [1–3]. Annually, it was associated with approximately 2000 hospitalizations and five fatalities in children [4, 5], and societal costs of about 150 million EUR [1]. Routine, publicly funded varicella vaccination was introduced in Germany in 2004 for all children aged 11–14 months. In 2009, administration of a second dose to children aged 15–23 months was recommended [6, 7]. In 2004, two monovalent varicella vaccines were available, initially with a single-dose application schedule. A tetravalent measles-mumps-rubella-varicella (MMRV) vaccine with a two-dose schedule was licensed in Germany already in 2006, but was not regularly used in all parts of Germany before the recommendation of the second varicella dose. This was due to heterogeneous reimbursement regulations in the 16 federal states of Germany before 2009, with seven states (including Bavaria) covering only one-dose varicella vaccination as recommended at that time and, hence, largely excluding the use of two-dose MMRV [8].

Measles is another highly infectious, vaccine-preventable childhood disease with a potentially serious outcome: in the year 2000, an estimated 535,000 children died of measles worldwide [9]. The average costs per measles case in Germany were estimated as 373 EUR per outpatient and 1877 EUR per inpatient, with incidence and hospitalization rates varying widely across years (from <0.1 to 38.9 per 100,000 inhabitants per year and 2%–40%, respectively) [10]. Routine vaccination against measles had already been introduced in Eastern Germany in 1970 and in Western Germany in 1973 [11, 12]. It is routinely administered by using a combined MMR or MMRV vaccine for all children aged 11–14 months, with a second dose at 15–23 months of age. The first dose may be administered at an earlier age (9 months), if necessary due to the epidemiological situation.

Varicella and measles vaccinations are voluntary in Germany and offered to parents free of costs. Vaccination of children is not a routine requirement for attendance at a public child daycare unit or school/university. Only private childcare units can introduce restricted access for unvaccinated children, but the public sector (including schools and universities) can deny admission to unvaccinated children/adolescents only in exceptional cases (e.g. in the case of an outbreak) [13, 14].

The World Health Organization (WHO) initially recommended universal varicella vaccination only for those countries where vaccination rates of at least 85% can be achieved and sustained, as a lower coverage may potentially be associated with a higher risk of complications due to an age shift of varicella infections to higher age groups [15]. This recommendation was replaced in 2014, and varicella vaccination coverage of at least 80% is now endorsed [16]. With regard to measles, vaccination coverage of at least 95% is considered necessary to eliminate the disease, however, this target has still not been reached in all regions of Germany [12, 17–20].

Routine surveillance of vaccination coverage is not implemented in Germany. Vaccination rates are usually assessed from school entrance health examinations, health claim data or population surveys [21], as well as from practices using a specific electronic vaccination scheduling program [22]. Studies investigating varicella and/or measles vaccination showed substantial differences in coverage rates between the various federal states of Germany [20, 23–26].

Previous investigations in the region of Munich in the federal state Bavaria had shown that varicella vaccination coverage (first dose) in children 18–36 months of age had increased from 38% in 2006 to 68% in 2011, whereby measles vaccination coverage (first dose) had shown a level of 87% to 91% [27–29]. However, vaccination rates and associated socio-demographic factors in the predominantly urban, economically-favored Munich region may differ from other Bavarian regions, and the socio-demographic factors that impact upon parental decisions concerning vaccination may differ for varicella and measles vaccination. Hence, the main objective of the present analysis was to compare varicella and measles vaccination rates from Munich with a more rural area, and to investigate determinants of acceptance both for varicella and measles vaccination.

## Methods

### Study design

Data were collected from two regions in Bavaria, each consisting of a major city and its surrounding regions (administrative districts). Munich City is the capital of Bavaria, situated in the south of this federal state. With its surrounding districts, the Munich region is of a largely urban character, covering 1,326,807 inhabitants from Munich City and 317,543 from the surrounding districts in the year 2008. Würzburg City is situated about 250 km north of Munich and is the capital of a region in Northern Bavaria, surrounded by a large rural area of administrative districts comprised of villages and small towns. Würzburg covers 133,501 inhabitants from Würzburg City and 160,273 from the surrounding districts [30]. All referrals to Munich or Würzburg in this study include the respective city with the surrounding administrative districts.

An extensive description of the data collection methods was provided by Streng (2010) [27] and Hagemann (2016) [29]. Random samples of 600 children aged 18–36 months were drawn per study region (Munich, Würzburg) and per survey year (2009, 2010, 2011) from the registration offices in the Munich and Würzburg regions (3600 children in total). Parents received information about the study per postal mail, including an informed consent form and a questionnaire on socio-economic variables, the varicella and measles history and the vaccination status of the child. Additionally, the questionnaire collected information on parental information status and on the recommendation by the child’s physician (usually a pediatrician, sometimes a general practitioner/family doctor) regarding the more recently introduced varicella vaccination. The parents also received a prepaid return envelope and a booklet for the child as an incentive.

Ethical approval was obtained from the Ethics Committees of the Medical Faculties at the University Hospitals in Munich and Würzburg.

### Statistical analysis

Returned, valid questionnaires were entered into a MS Access 2003 database and the statistical analysis was performed using IBM SPSS 22.0 / 23.0. Data was analyzed descriptively and reported as percentages or median values with inter-quartile range (IQR), referring to the actual data available per variable. Data was compared using Pearson’s Chi^2^-test or Fisher’s Exact Test for categorical data, and Mann-Whitney U-test for continuous data, with a two-sided significance level of *p* < 0.05. For bivariate as well as for multivariable logistic regression analyses, data collected during the three survey years were pooled for each region.

Varicella vaccination rates (1st dose) for each region and survey year were assessed for children who had been susceptible (i.e., no previous varicella vaccination/history) at the age of 11 months [27, 29]. Measles vaccination rates were assessed for all participating children, as the number of children with previous measles was very low and, therefore, had no relevant impact on the results.

Factors potentially associated with the parental acceptance of varicella or measles vaccination were identified in bivariate analyses for each region, with varicella or measles vaccination status (vaccinated yes/no) at time of the survey being used as a dependent variable. Odds ratios (OR) and 95% confidence intervals (CI) were calculated for each potential determinant. The school education levels of the mother and father were strongly correlated and were therefore combined in the variable ‘highest possible school education of at least one parent’ for the regression analyses.

Multivariable logistic regression models were built for both measles and varicella vaccination to further investigate the determinants of parental acceptance of vaccinations. Due to large socio-economic differences in the study populations in Munich and Würzburg, separate analyses were performed for each region. The model building approach for each region and vaccination was an automated stepwise forward selection process using Likelihood ratios and a 10%-significance level as entry criterion. For measles vaccination, factors which were solely collected for varicella vaccination were excluded from the analyses (i.e. recommendation of varicella vaccination by the physician).

Kaplan-Meier failure curves using age at vaccination as event time and age at survey or – where relevant – age at disease as censoring times were computed for both regions and vaccinations [27].

## Results

### Response rates and socio-demographic characteristics of participants

From 600 questionnaires sent out for each survey year and region, valid questionnaires were obtained from Munich for 330 (55%; 2009), 301 (50%; 2010) and 302 (50%; 2011) children, and from Würzburg for 356 (59%; 2009), 370 (62%; 2010) and 344 (57%; 2011) children. Five questionnaires from Würzburg were excluded due to the age of the child at first varicella vaccination exceeding 36 months of age. Thus, data from a total of 1998 children were analyzed.

Table 1 summarizes the socio-demographic characteristics of the study populations in Munich (*n* = 933) and Würzburg (*n* = 1065). In Munich, a substantially higher proportion of participants lived in an urban region (77% compared with 43% in Würzburg; *p* < 0.001). The proportion of parents with the highest possible school education (university entrance level) was higher in Munich (mothers 56%, fathers 59%) compared to Würzburg (mothers 43%, fathers 41%; for both *p* < 0.001). Comparison of household characteristics showed a smaller size of residence (median 92 m^2^ vs. 120 m^2^, p < 0.001), a lower number of persons per household (≥4 persons: 52% vs. 70%, p < 0.001) and a lower proportion of children visiting a child daycare unit (65% vs. 76%, *p* < 0.001) for Munich than for Würzburg, respectively.

**Table 1:** Demographic and socioeconomic characteristics of participants in Munich and Würzburg in 2009–2011; data from the three survey years pooled for each region

	Munich (2009–2011) (N tot. = 933)		Würzburg (2009–2011) (N tot. = 1065)		
	N	n (%), or median (IQR)	N	n (%), or median (IQR)	*p*-value^c^
Demographic characteristics – child					
Gender female; n (%)	933	463 (49.6)	1065	519 (48.7)	0.720
Nationality German^a^; n (%)	930	898 (96.6)	1058	1048 (99.1)	<0.001***
Country of birth Germany; n (%)	882	864 (98.0)	1022	1.005 (98.3)	0.609
Inhabitant of urban region; n (%)	933	714 (76.5)	1065	460 (43.2)	<0.001***
Demographic characteristics – parents					
Age of mother (y); median (IQR)	912	35.0 (32.0–39.0)	1040	35.0 (31.3–38.0)	0.069
Highest possible school education mother^b^; n (%)	920	515 (56.0)	1052	456 (43.3)	<0.001***
Highest possible school education father^b^; n (%)	860	507 (59.0)	1013	415 (41.0)	<0.001***
Highest possible school education^b^, at least one parent; n (%)	923	637 (69.0)	1056	587 (55.6)	<0.001***
Household characteristics					
Size of residence (m^2^); median (IQR)	918	91.5 (76.0–120.0)	1044	120.0 (90.0–144.3)	<0.001***
More than 3 persons living in residence; n (%)	925	478 (51.7)	1062	739 (69.6)	<0.001***
Attendance of child-care unit (>10 h/week); n (%)	930	603 (64.8)	1061	810 (76.3)	<0.001***
Statutory health insurance; n (%)	924	653 (70.7)	1056	788 (74.6)	0.054

^a^including cases of dual nationality (German and other)

^b^university entrance diploma at end of grammar school (‘Abitur’)

^c^Pearson’s Chi^2^ test and Mann-Whitney-U-test, respectively

***: significant at 0.1% level

### Varicella history and vaccination coverage

A history of varicella infection was reported in 6.9% of Munich children and in 5.0% of Würzburg children (*p* = 0.085, Table 2). Children in Munich were significantly younger at the age of varicella infection than in Würzburg (median age 15 vs. 20 months, *p* = 0.010). Across all survey years, 885 of 933 (95%) children in Munich and 1019 of 1065 (96%) children in Würzburg had been susceptible for varicella vaccination at the age of 11 months.

**Table 2:** Varicella and measles history and vaccination status of children in Munich and Würzburg; data from the three survey years pooled for each region

	Munich (N tot. = 933)		Würzburg (N tot. = 1065)		
	N	n (%), or median (IQR)	N	n (%), or median (IQR)	*p*-value^a^
Varicella history (all children)					
History of any previous varicella infection; n (%)	933	64 (6.9)	1065	53 (5.0)	0.085
- If yes, age at varicella infection (mo.); median (IQR)	64	14.5 (6.5–20.8)	53	20.0 (10.5–31.5)	0.010*
Varicella vaccination status (children susceptible for varicella at 11 months of age, first dose)					
Vaccinated against varicella at time of survey; n (%)	885	555 (62.7)	1019	802 (78.7)	<0.001***
- If yes, age at varicella vaccination (mo.); median (IQR)	555	12.5 (11.8–13.9)	802	12.5 (11.7–14.3)	0.891
- If yes, vaccine type; n (%)	549		800		0.090
monovalent		103 (18.8)		182 (22.8)	
tetravalent		446 (81.2)		618 (77.3)	
Varicella vaccination recommended by physician; n (%)	868	578 (66.6)	1009	820 (81.3)	<0.001***
Measles history (all children)^c^					
History of any previous measles infection; n (%)	932	2 (0.2)	1063	2 (0.2)	0.560
- If yes, age at measles infection; per case (mo.)	2	12; 21	2	36^b^	–
Measles vaccination status (all children, first dose)^c^					
Vaccinated against measles at time of survey; n (%)	931	833 (89.5)	1065	994 (93.3)	0.002**
- If yes, age at measles vaccination (mo.); median (IQR)	820	12.0 (11.0–14.0)	985	12.0 (11.0–14.0)	0.088

^a^Pearson’s Chi^2^ test (Fisher’s exact test in cases with expected number < 5 in more than 20% of cells) and Mann-Whitney-U-test, respectively

^b^the age at measles infection of the other child is unknown

^c^Due to the very low numbers of children with preceding measles infection, measles vaccination rates were reported for all children. For single children, data on measles history, measles vaccination status or age at measles vaccination was not available

***: significant at 0.1% level, **: significant at 1% level, *: significant at 5% level

**Fig. 1:**
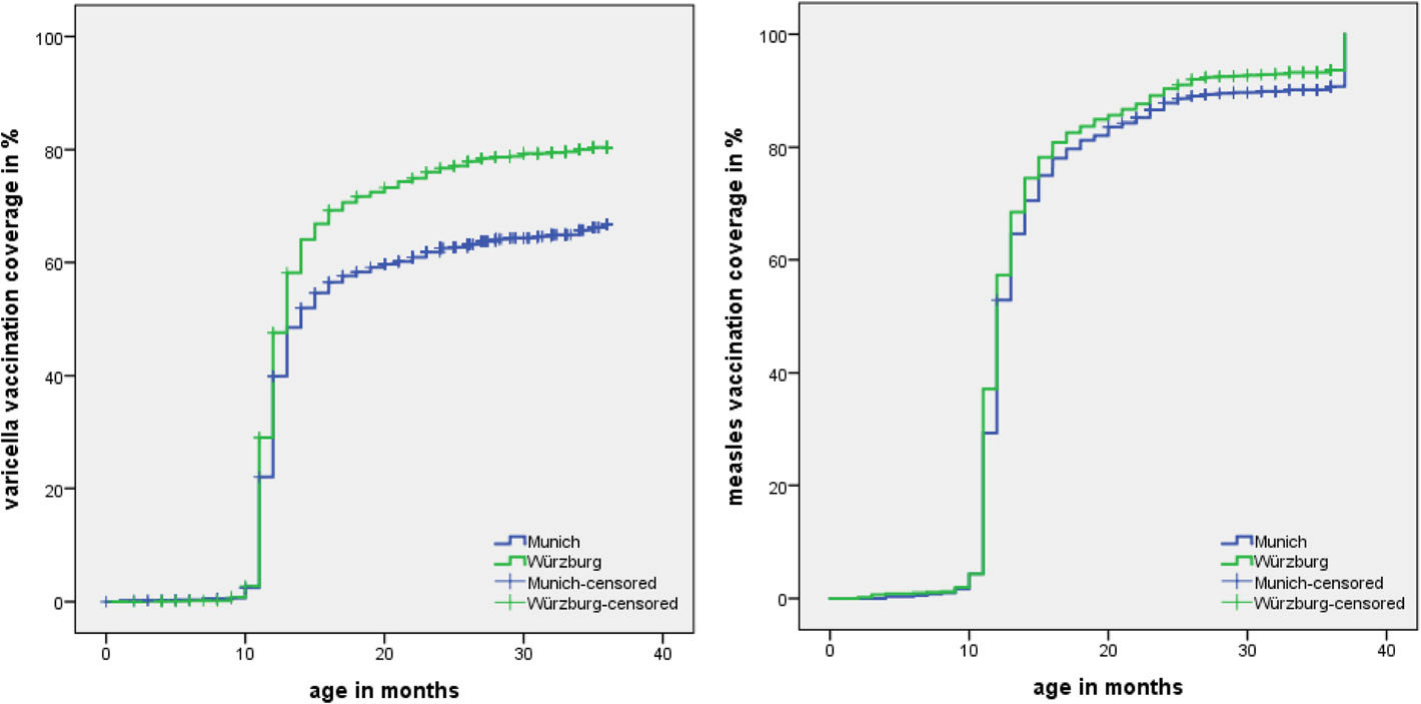
Varicella and measles vaccination rates (1st dose) by age and by area (Munich, Würzburg). Data from all three survey years (2009–2011) pooled for each region. Kaplan-Meier failure curves using age at varicella/measles vaccination as event time and age at survey or – where relevant – age at varicella/measles disease onset as censoring times. Varicella vaccination data from Munich as in [29]

Overall (2009–2011) varicella vaccination coverage (first dose) in susceptible children was 63% in Munich and 79% in Würzburg. In Munich, coverage increased from 53% in 2009 to 69% in 2011, and in Würzburg from 72% in 2009 to 83% in 2011, with significantly higher coverage in Würzburg for each survey year (*p* < 0.001 in all years, Table 3). Age at varicella vaccination was similar in both regions (median 12.5 months; Table 2); vaccination rates by age and study region are provided in Fig. 1.

**Table 3:** Varicella and measles vaccination coverage, by region and survey year

		Survey 2009			Survey 2010			Survey 2011		
		N	n (%)	*p*-value^a^	N	n (%)	*p*-value^a^	N	n (%)	*p*-value^a^
Vaccinated against varicella^b^at time of survey, first dose; n (%)	Munich	314	165 (52.5)	<0.001***	288	194 (67.4)	<0.001***	283	196 (69.3)	<0.001***
	Würzburg	340	246 (72.4)		350	283 (80.9)		329	273 (83.0)	
Varicella vaccination recommended by physician^b^	Munich	304	183 (60.2)	<0.001***	282	188 (66.7)	<0.001***	282	207 (73.4)	<0.001***
	Würzburg	335	255 (76.1)		346	285 (82.4)		328	280 (85.4)	
Vaccinated against measles at time of survey, first dose; n (%)	Munich	328	289 (88.1)	0.162	301	269 (89.4)	0.094	302	275 (91.1)	0.039*
	Würzburg	354	324 (91.5)		368	343 (93.2)		343	327 (95.3)	

^a^Pearson’s Chi^2^ test

^b^only children susceptible for varicella at 11 months of age

***: significant at 0.1% level, *: significant at 5% level

Varicella vaccination had been less frequently recommended by physicians in Munich than in Würzburg (all susceptible children, 67% vs. 81%, p < 0.001; Table 2; for change over time in both regions cf. Table 3). Recommendation of the vaccination was higher in children visiting a child daycare unit (Munich: 67%, *p* = 0.074, Würzburg 78%, *p* = 0.010) compared to children not visiting a daycare unit. Almost all parents stated that they were aware of the fact that children can be vaccinated against varicella (96% of the parents in Munich and 97% of parents in Würzburg).

### Measles history and vaccination coverage

Overall, four children had a history of measles (at ages 12, 21 and 36 months and, in the case of one child, the age at infection was unknown) (Table 2).

Overall measles vaccination coverage (first dose) was 90% in Munich and 93% in Würzburg (*p* = 0.002, Table 2). In both regions, coverage slightly increased from 2009 to 2011. Coverage levels were not significantly different between Munich and Würzburg in 2009 and 2010, but were lower in Munich in 2011 (91% vs. 95%; *p* = 0.039; Table 3). Age at measles vaccination did not differ between the regions (median age 12 months; Table 2); for vaccination rates by age and region see Fig. 1.

Almost all children that had received a vaccination against varicella were also vaccinated against measles, except one child each in Munich and Würzburg. In contrast, only 77% of all children vaccinated against measles were also vaccinated against varicella.

### Determinants for parental acceptance of varicella and measles vaccination

#### Factors associated with varicella vaccination

The most influential factor associated with varicella vaccination in both regions in bivariate analyses was the recommendation of this vaccination by the physician (Table 4). This association was lower in Munich (OR 18.8; 95%CI 13.2–26.8) than in Würzburg (OR 35.9; 95%CI 23.6–54.5). Attendance at a child daycare unit was also significantly associated with varicella vaccination in both regions (Munich: OR 1.5; 95%CI 1.1–2.0; Würzburg OR 1.7; 95%CI 1.2–2.4). Acceptance of varicella vaccination increased significantly over time in both regions (for example, Munich: OR (2011 vs. 2009) 2.0; 95%CI 1.5–2.9; Würzburg: OR (2011 vs. 2009) 1.9; 95%CI 1.3–2.7). Older age of the mother (> 35 years) at the time of the survey was negatively associated with varicella vaccination in Munich (OR 0.7, 95%CI 0.5–0.9), but not in Würzburg (OR 0.8, 95%CI 0.6–1.1). No other socio-demographic factors investigated in the study were associated with this vaccination.

**Table 4:** Bivariate logistic regression analyses to assess factors associated with likelihood of varicella and measles vaccination; data from all three survey years pooled for each region

Bivariate logistic regression analyses (comparing children with and without varicella vaccination or measles vaccination, respectively^a^)	Varicella Vaccination		Measles Vaccination	
	Munich (2009–2011) OR (95% CI)	Würzburg (2009–2011) OR (95% CI)	Munich (2009–2011) OR (95% CI)	Würzburg (2009–2011) OR (95% CI)
	N^b^ = 885	N^b^ = 1019	*N* = 931	N = 1065
Age of mother >35 years vs. ≤ 35 years	0.65 (0.50–0.86)^f^	0.82 (0.61–1.12)	0.90 (0.59–1.38)	0.99 (0.60–1.62)
Gender of child female vs. male	1.30 (0.99–1.71)	1.14 (0.84–1.54)	0.94 (0.62–1.44)	1.17 (0.72–1.90)
Physician recommended varicella vaccination vs. no recommendation	18.79 (13.16–26.84)^e^	35.87 (23.62–54.47)^e^	–	–
Survey year				
2010 vs. 2009 (reference)	1.86 (1.34–2.60)^e^	1.61 (1.13–2.31)^f^	1.13 (0.69–1.86)	1.27 (0.73–2.21)
2011 vs. 2009 (reference)	2.03 (1.54–2.85)^e^	1.86 (1.28–2.71)^f^	1.37 (0.82–2.31)	1.89 (1.01–3.54)^g^
Attendance of childcare unit >10 h/week vs. no or less frequent attendance	1.50 (1.13–2.00)^f^	1.69 (1.21–2.35)^f^	2.00 (1.31–3.04)^f^	1.84 (1.10–3.06)^g^
Highest possible school education^c^ at least one parent	0.75 (0.55–1.01)	0.79 (0.58–1.08)	0.85 (0.53–1.35)	0.54 (0.32–0.91)^f^
Inhabitant of city vs. administrative district	0.73 (0.53–1.03)	0.96 (0.71–1.30)	0.76 (0.45–1.29)	1.18 (0.72–1.93)
Family size ≥4 persons vs. < 4 persons	0.85 (0.64–1.11)	0.80 (0.58–1.12)	1.04 (0.68–1.57)	0.83 (0.48–1.42)
Private health insurance vs. statutory health insurance	0.85 (0.63–1.42)	1.26 (0.88–1.81)	0.72 (0.47–1.12)	0.78 (0.46–1.33)

^a^: all vaccination data refers to first dose

^b^: only children susceptible at the age of 11 months

^c^: University entrance diploma at end of grammar school (‘Abitur’)

^e^: significant at 0.1% level

^f^: significant at 1% level

^g^: significant at 5% level

In adjusted logistic regression models for each region (Table 5), recommendation of varicella vaccination by the child’s physician remained the strongest predictor for the acceptance of the vaccination in both regions (Munich OR 19.7; 95%CI 13.6–28.6; Würzburg OR 34.7; 95%CI 22.6–53.2). The increasing acceptance of varicella vaccination over time was confirmed in the adjusted model (Munich OR (2011 vs. 2009) 1.6; 95%CI 1.0–2.5; Würzburg OR (2011 vs. 2009) 1.8; 95%CI 1.1–2.9). Attendance at a child daycare unit was positively associated with varicella vaccination in Munich (OR 1.5; 95%CI 1.1–2.2).

**Table 5:** Multivariable logistic regression analyses to assess factors associated with likelihood of varicella and measles vaccination, respectively; data from all three survey years pooled for each region

Multiple logistic regression analyses (comparing children with and without varicella vaccination or measles vaccination, respectively^a^)	Varicella Vaccination		Measles Vaccination	
	Munich (2009–2011) OR (95% CI)	Würzburg (2009–2011) OR (95% CI)	Munich (2009–2011) OR (95% CI)	Würzburg (2009–2011) OR (95% CI)
	N^b^ = 885	N^b^ = 1019	*N* = 931	*N* = 1065
Physician recommended varicella vaccination vs. no recommendation	19.71 (13.57–28.63)^e^	34.66 (22.59–53.18)^e^	N/A^c^	N/A^c^
Survey year				
2010 vs. 2009 (reference)	1.68 (1.08–2.60)^g^	1.59 (0.98–2.56)	–	1.09 (0.61–1.93)
2011 vs. 2009 (reference)	1.60 (1.03–2.47)^g^	1.78 (1.08–2.94)^g^	–	2.01 (1.03–3.90)^g^
Attendance of childcare unit >10 h/week vs. no or less frequent attendance	1.52 (1.05–2.22)^g^	–	1.97 (1.28–3.03)^f^	1.78 (1.03–3.10)^g^
Highest possible school education^d^ at least one parent	–	–	–	0.50 (0.29–0.85)^g^
Private health insurance vs. statutory health insurance	–	–	0.64 (0.41–1.01)	–

Model building approach was an automated forward-selection procedure using Likelihood ratios and a 10%-significance level as entry criterion. Only variables that fulfilled the entry criterion are displayed

^a^: all vaccination data refers to first dose

^b^: only children susceptible at the age of 11 months

^c^: only collected regarding recommendation of varicella vaccination

^d^: university entrance diploma at end of grammar school (‘Abitur’)

^e^: significant at 0.1% level

^f^: significant at 1% level

^g^: significant at 5% level

#### Factors associated with measles vaccination

In bivariate analyses, the attendance at a child daycare unit was positively associated with measles vaccination in both regions (Munich: OR 2.0; 95%CI 1.3–3.0; Würzburg: OR 1.8; 95%CI 1.1–3.1, Table 4). In Würzburg, a slight increase in measles vaccination coverage over time was observed after comparing 2011 with 2009 (OR 1.9; 95%CI 1.01–3.5). A higher school education (‘Abitur’, corresponding to general qualification for university entrance) of at least one parent was negatively associated with measles vaccination in Würzburg (OR 0.5, 95%CI 0.3–0.9).

In the adjusted model, attendance at a childcare unit was confirmed as the factor with the highest influence on likelihood of measles vaccination in both regions (Munich OR 2.0; 95%CI 1.3–3.0; Würzburg OR 1.8; 95%CI 1.0–3.1, Table 5). The measles vaccination rates increased between 2009 and 2011 in Würzburg (OR (2011 vs. 2009) 2.0; 95%CI 1.0–3.9). A higher school education of at least one parent (‘Abitur’) was negatively associated with measles vaccination in the multivariable model in Würzburg (OR 0.5; 95%CI 0.3–0.9).

## Discussion

The objective of the present analysis was to compare varicella and measles vaccination rates and factors associated with the acceptance of vaccination in two Bavarian regions based on parent surveys in 2009–2011.

The results in Munich showed an increasing coverage of varicella vaccination – first recommended in 2004 - from 53% in 2009 to 69% in 2011 [29]. In Würzburg, the vaccination coverage increased similarly, but was about 15–20% points higher (72% in 2009, 83% in 2011) than in Munich. Although the varicella vaccination rates increased over the study period, the initial WHO-defined goal of at least 85% varicella vaccination coverage still had not yet been reached in both regions as of 2011.

The vaccination coverage for measles, first recommended in 1970 (Eastern Germany) and in 1973 (Western Germany) [11] was substantially higher than for varicella in both study regions, even though still below the WHO target of 95% vaccination coverage. Nevertheless, during the three-year observation period, the already high measles vaccination rates further increased by 3 % points for the first dose in Munich and in Würzburg. The WHO goal of 95% measles vaccination coverage was reached in Würzburg in 2011, whereas vaccination rates were still below this target in Munich (91% in 2011) in the investigated age group from 18 months up to 3 years.

Nevertheless, school entry health examinations three years later (2014) on children aged about 6 years showed that the initial differences in measles vaccination coverage between both regions observed in the present study had disappeared, with coverage between 97 and 98% in both regions at the time of school entry (for both cities as well as their surrounding districts) [31]. This increase indicates the success of national efforts and campaigns to enhance measles vaccination coverage. However, compliance with the recommended age for vaccination was still considerably delayed and suboptimal in Munich, with the risk of outbreaks in pre-school children. Delayed vaccinations and potential reasons have been discussed by Goffrier et al. (2016) and included lack of time, postponed or cancelled physician appointments [32].

The positive attitude of physicians and other healthcare professionals towards vaccinations – and consequently the recommendation of vaccination - has been shown to be an important determinant of vaccination coverage [27, 33–36]. In Germany, children of toddler age are usually vaccinated in pediatric practices; thus, pediatricians are the primary persons who can inform and reassure parents with concerns about the meaningfulness and safety of vaccinations [8]. In our study, recommendation of the more recently introduced varicella vaccination by the child’s physician was the only independent factor consistently and significantly associated with a parental decision to vaccinate their child against varicella in Munich [27, 29] and in Würzburg. Interestingly, differences in the strength of the association were seen in the bivariate and multivariable logistic regression models, indicating a stronger compliance of parents towards the recommendation of the physician in Würzburg. Parents in Munich appear to behave more independently of the physician’s opinion, which might be associated with a higher proportion of parents with higher educational level in comparison to the Würzburg area. A more critical attitude towards vaccinations in well-educated parents has been described in some studies [32, 37, 38], while others found no impact or even a positive impact of a high education level on vaccination uptake [36, 38–40]. Thus, strategies to increase acceptance of varicella vaccination may need to be adapted by country, by region, and according to the educational level of the parents.

Although the rate of varicella vaccination recommendation by the physicians increased in both regions, some of the comments provided by parents on the questionnaire indicated a negative attitude by some physicians, mostly based on missing knowledge regarding varicella vaccination and the strategy implemented by the Standing Committee on Immunization (STIKO) in Germany. Such comments included, for example: “Our physician recommends the varicella vaccination at school entrance”, “Only measles vaccination recommended”, “According to our pediatrician, varicella vaccination increases cases of shingles after vaccination despite the argumentation of the vaccination authorities of preventing them”, “Second varicella vaccination is not necessary”. Lack of knowledge regarding illness/vaccination by the vaccinating physicians themselves and lack of adequate information about vaccination provided for the parents by healthcare professionals had been identified as factors contributing to vaccination hesitancy and low vaccination uptake for other vaccines [35, 36, 40, 41]. The key reason for hesitancy of both physicians and parents was fear of adverse side effects and vaccine safety concerns [35, 40]. It seems essential that the continuing education of pediatricians and other healthcare professionals as the most influential source of vaccination information is further optimized to further increase varicella vaccination rates, as suggested for other vaccines [24, 35, 37, 41]. This can only be achieved by providing continuous education on the objectives, the safety and the efficacy of varicella vaccination, and may be supported by training on physician communication strategies to improve the dialogue with vaccine-hesitant parents [35, 36, 40, 42].

The influence of socio-demographic factors on the acceptance of vaccinations has been investigated in many studies [33, 36, 38–40, 43]. Interestingly, in our survey conducted during the years 2009 to 2011, attendance at child daycare units strongly influenced the likelihood of both varicella and measles vaccination. In 2008, Germany introduced a new law (Childcare Funding Act; “Kinderförderungsgesetz” [44]), which allowed parents a legal right of access to daycare facilities for their children. The law aimed at increasing the number of the hitherto limited places for children in such facilities, as child daycare is regarded a necessary precondition if both parents wish to take up an employment. Indeed, the new law resulted in an increased availability of these childcare facilities in the years that followed, with a subsequent increase in the proportion of children visiting such facilities [45]. In the Munich surveys, the proportion of children up to 36 months of age attending childcare units increased from 45%–54% in 2006–2008 to 59%–67% in 2009–2011 [27, 29]. This may have contributed indirectly to the increased vaccination coverage rates, as the risk of contracting measles or varicella in this age group is likely to be higher in daycare environments compared to home, and working parents may wish to reduce this risk by vaccination, also to avoid time off work in the event of the child’s disease [46]. Accordingly, varicella vaccination was more often recommended by physicians for children who visited a daycare unit compared to children who did not. Although only private daycare institutions may insist on vaccinations as a precondition for attendance, parents preferring specific public childcare facilities (e.g., close to home or the working place) may be more willing to comply with the vaccination suggestions provided by these facilities to increase the chance for their child being admitted there.

The increase of varicella vaccination rates was probably also influenced by the more widespread use of the combined MMRV vaccine since 2009 [47]. As vaccination against measles/mumps/rubella (MMR) was already well established, adding the varicella component to the MMR vaccine instead of applying varicella vaccine in a separate injection appeared to further enhance varicella vaccination coverage (without a negative impact thus far on coverage for measles) [24, 29].

The generally higher acceptance of measles vaccination and the somewhat delayed introduction of MMRV in Bavaria may also explain the observation that in our survey almost all children vaccinated against varicella were vaccinated against measles, but not all children vaccinated against measles were vaccinated against varicella. The lower acceptance of varicella vaccination in both study areas was further confirmed when the STIKO changed the recommendation in late 2011 towards separate first-dose vaccination for MMR and for varicella, due to a slightly increased risk of febrile seizures observed for first-dose MMRV vaccination [48]. This change resulted in a decline of varicella vaccination in both study areas, whereas measles vaccinations remained stable [48]. However, such an impact of the change in recommendation was not observed in other regions of Germany [22, 49].

Initially, vaccination coverage for varicella differed substantially between the federal states due to the different reimbursement regulations [8], and still differed in annual preschool entrance health examinations of children 5–6 years of age in 2014 (first-dose coverage between 69% and 95%; Bavaria 76%) [26]. Overall measles vaccination coverage in preschool children was generally higher and varied only slightly between the federal states (between 95% and 98%) [26]. However, between and within the federal states measles vaccination rates from health insurance claims data for 2017 still showed substantial regional differences in children under the age of two years, with usually lower vaccination coverage in the more southern federal states, including Bavaria [20].

Regional differences in the distribution of health care providers with a critical attitude towards vaccinations (e.g., homeopaths, alternative practitioners) have been mentioned as potential reasons for the observed regional differences in vaccination coverage [32]. Higher parental educational level potentially associated with a more critical view on vaccinations and, as a second point, less frequent attendance at a child daycare unit may also provide a potential explanation for the heterogeneity in vaccination coverage between north and south of Germany and also between the regions within Bavaria [32].

Strengths and limitations of the overall project were discussed previously [27, 29]. One of the strengths of the present analysis is the availability of comparable data within two regions with different socio-economic characteristics of the study population. The study was performed simultaneously in Munich and Würzburg over three consecutive years, allowing a direct comparison of the regions. A limitation of the present analysis on measles was the lack of information regarding the explicit recommendation of this vaccination by physicians. However, measles vaccination has been well established in the German population for several decades. Hence, it can be assumed that in contrast to the recently introduced varicella vaccination, parental acceptance for measles vaccination was more dependent from the parents’ own judgement and less dependent from physician’s recommendation. Nevertheless, regional differences could also be confirmed for measles vaccination, although much less pronounced than for varicella vaccination.

## Conclusions

Varicella and measles vaccination rates in children aged 18 to 36 months differed between regions, with consistently higher rates observed in the Würzburg region of Northern Bavaria. Within each region, vaccination coverage was lower for varicella than for measles. Vaccination coverage clearly increased during the observation period in both regions and for both vaccinations. However, vaccination rates in the age group under investigation were still insufficient to prevent outbreaks.

The regions showed substantial differences between the study populations regarding socio-demographic factors. Different factors determined parental acceptance of varicella and measles vaccination. Measles vaccination status in the investigated age group was mainly dependent upon the attendance at a childcare unit and on the educational levels of the parents. In contrast, for the more recently introduced varicella vaccination recommendation by the physician had the strongest impact. This emphasizes the need for regional and vaccination-specific programs to increase vaccination coverage and to improve compliance in relation to the timeliness of the vaccinations.

## Abbreviations

CI: Confidence Interval
IQR: Inter-quartile range
MMR(V): Measles Mumps Rubella (Varicella)
OR: Odds Ratio
STIKO: Standing Committee on Immunization
WHO: World Health Organization
